# Relationship of Neural Correlates of Gait Characteristics and Cognitive Dysfunction in Patients with Mild Cognitive Impairment

**DOI:** 10.3390/jcm12165347

**Published:** 2023-08-17

**Authors:** Yeo Jin Kim, Ingyu Park, Hui-Chul Choi, Moo-Eob Ahn, Ohk-Hyun Ryu, Daehun Jang, Unjoo Lee, Sang-Kyu Lee

**Affiliations:** 1 Department of Neurology, Kangdong Sacred Heart Hospital, Seoul 05355, Republic of Korea; yjhelena@hanmail.net; 2 Department of Electronic Engineering, Hallym University, Chuncheon 24252, Republic of Korea; qkrdlsrb3946@naver.com (I.P.); deahun123@naver.com (D.J.); 3 Department of Neurology, Hallym University-Chuncheon Sacred Heart Hospital, Hallym University College of Medicine, Chuncheon 24253, Republic of Korea; dohchi@hallym.or.kr; 4 Department of Emergency Medicine, Hallym University-Chuncheon Sacred Heart Hospital, Hallym University College of Medicine, Chuncheon 24253, Republic of Korea; mooeob@gmail.com; 5 Division of Endocrinology and Metabolism, Department of Internal Medicine, Hallym University-Chuncheon Sacred Heart Hospital, Hallym University College of Medicine, Chuncheon 24253, Republic of Korea; ohryu30@gmail.com; 6 Division of Software, School of Information Science, Hallym University, Chuncheon 24252, Republic of Korea; 7 Department of Psychiatry, Hallym University-Chuncheon Sacred Heart Hospital, Hallym University College of Medicine, Chuncheon 24253, Republic of Korea

**Keywords:** gait, cognitive function, mild cognitive impairment, neural correlates

## Abstract

***Background***: Some patients with mild cognitive impairment (MCI) experience gait disturbances. However, there are few reports on the relationship between gait disturbance and cognitive function in patients with MCI. Therefore, we investigated the neural correlates of gait characteristics related to cognitive dysfunction. ***Methods***: Eighty patients diagnosed with MCI from three dementia centers in Gangwon-do, Korea, were recruited for this study. We defined MCI as a Clinical Dementia Rating global score of 0.5 or higher, with a memory domain score of 0.5 or greater. The patients were classified as having either higher or lower MMSE and the groups were based on their Mini Mental Status Examination z-scores. Multiple logistic regression analysis was performed to examine the association between the gait characteristics and cognitive impairment. Analyses included variables such as age, sex, years of education, number of comorbidities, body mass index, and height. ***Results***: Gait velocity, step count, step length, heel-to-heel base support, swing and stance phase duration, and support time were associated with cognitive function. A decrease in gray matter volume in the right pericalcarine area was associated with gait characteristics related to cognitive dysfunction. An increase in the curvature of gray matter in the right entorhinal, right lateral orbitofrontal, right cuneus, and right and left pars opercularis areas was also associated with gait characteristics related to cognitive dysfunction. ***Conclusion***: Since gait impairment is an important factor in determining activities of daily living in patients with mild cognitive impairment, the evaluation of gait and cognitive functions in patients with mild cognitive impairment is important.

## 1. Introduction

Mild cognitive impairment (MCI) is a state in which cognitive decline is present but activities of daily living (ADL) are minimally affected and independent living is possible [1]. MCI is the prodromal stage of dementia, which progresses to dementia in 10–15% of cases each year [2]. Although MCI is a disease in which cognitive decline is the main symptom of MCI, motor functions such as gait also play an important role in determining the ADL of patients with cognitive impairment [3]. In particular, because an altered gait may cause falls, it can lead to physical disability and admission to a nursing facility [4]. Additionally, falls due to gait deterioration can cause immobility, resulting in a rapid decline in cognitive function and poor prognosis in patients with cognitive impairment [5].

Patients with impaired cognitive function exhibit lower motor function when performing tasks that require cognitive function. In addition, when performing a complex task instead of a simple one, motor function deteriorates [6]. This means that gait requires cognitive functions, such as executive function and attention, rather than being a simple automated motor activity that uses minimal cognitive input [7]. Previous studies have shown that, not only in dementia patients but also in patients with MCI, a decline in cognitive function can lead to a deterioration in gait function [8]. Moreover, there have been attempts to indirectly measure cognitive function through motor function assessments, given that cognitive function reflects the motor phenotype. In these studies, gait speed and gait variability have been shown to reflect cognitive function [9,10].

Cognitive decline is caused by neuronal loss in the cerebral cortex, and the decline in cerebral cortical function due to specific brain atrophy might affect not only cognitive but also motor functions. In patients with MCI, a faster gait speed has been shown to be associated with an increase in left frontal cortex volume. An increase in dual-task gait speed has been linked to gray matter increases in various brain areas such as the medial and superior frontal gyrus, cingulate, precuneus, fusiform gyrus, middle occipital gyrus, inferior, and middle temporal gyrus [11]. However, most of these studies have primarily compared cognitively healthy patients with those having MCI. If motor function reflects cognitive function, identifying indicators within MCI that reflect cognitive function could be valuable. Although MCI is a heterogenous disease, understanding which characteristic indicators reflect deteriorating cognitive function could be instrumental in monitoring whether MCI will progress to dementia in the future. According to previous research, changes in gait function were also related to changes in CSF tau, an indicator of neurodegeneration in dementia [12]. This would be also helpful in assessing and treating patients if there are indicators to recognize cognitive function within MCI without conducting a cognitive function test. Additionally, while no distinct brain area related to gait function has been discovered in cognitively healthy subjects from previous studies, a high correlation between gait speed and the frontal area was found in patients with MCI [13]. In a state where cognitive function is normal, gait problems can occur due to other physical changes rather than changes in the brain. However, in a group with cognitive changes like MCI, it could be inferred that changes in gait originate from changes in brain structure. Nevertheless, this study did not examine gait parameters related to cognitive function.

Therefore, we aimed to explore the gait parameters related to cognitive function within the MCI patients and to examine the brain areas that have undergone changes in relation to these gait parameters.

## 2. Methods

### 2.1. Participants

We prospectively recruited 80 patients with mild cognitive impairment at Chuncheon Sacred Heart Hospital between October 2020 and April 2021. The inclusion criteria were age between 40 and 100 years, a diagnosis of minor neurocognitive disorder as determined by the Diagnostic and Statistical Manual of Mental Disorders Fifth Edition (DSM-V), absence of dementia according to physicians’ judgment, and a Clinical Dementia Rating (CDR) global score of 0.5 with a memory domain score of 0.5 or greater. However, the MCI subtypes were not examined. Exclusion criteria included severe illness with an anticipated fatal outcome within three months, language barrier, deafness or blindness, and an inability to provide informed consent. We also excluded from the study patients with disabilities observed during the physical exam that could impact gait function. The groups were classified into two groups: Higher MMSE (H-MMSE) subgroup which showed performance on the Mini Mental Status Examination (MMSE) of less than 1.5 standard deviation (SD) below the normative mean, while the Lower MMSE (L-MMSE) subgroup showed performance on the MMSE of more than 1.5 SD below the normative mean. 

Written informed consent was obtained from each patient, and the Institutional Review Board of Chuncheon Sacred Heart Hospital approved the study protocol (IRB number: Chuncheon 2020-09-005).

### 2.2. Clinical Assessment

We recorded demographics, including age, sex, and years of education, and measures of body composition, including height, weight, and waist circumference. We also checked the blood pressure and blood test results, including fasting glucose and total cholesterol levels. Patients completed the depression scale (Short form of Geriatric Depression Scale, SGDS), anxiety scale (Korean Geriatric Anxiety Inventory, K-GAI), quality of life scale (Geriatric Quality of Life Dementia, GQOL-D), and the Korean National Health and Nutrition Examination Survey (KNHANES). Additional medical comorbidities were checked during the clinic interviews. 

### 2.3. Gait Assessment

The GAITRite^®^ instrumentation (CIR systems Inc., Havertown, PA, USA) comprises an electronic walkway measuring 5.6 m in length and 0.9 m in width. Each patient was instructed to walk at a normal pace without a gait aid on the walkway, and each patient walked on the GAITRite pad in a single pass. The study coordinator observed each patient’s gait without any interference. For this analysis, we focused on the following gait variables: gait velocity (m/s), cadence (number of steps per minute), step time (s), step length (cm), cycle time (s), heel-to-heel (H-H) base support (cm), swing percentage of the cycle phase (%), stance percentage of the cycle phase (%), double support percent cycle phase (%), step time variability (%), and step length variability (%). Variability was expressed as the coefficient of variation, which was calculated as the standard deviation of the step time or step length divided by the mean of the step time or step length, multiplied by 100%.

### 2.4. MRI Acquisition

Standardized T1, T2, fluid-attenuated inversion recovery (FLAIR), and three-dimensional (3D) T1-weighted images were acquired from all eligible participants at Chuncheon Sacred Heart Hospital using the same 3.0T MRI scanner (Siemens Skyra). Next, 3D T1-weighted structural brain images were acquired using a Magnetization Prepared Rapid Acquisition Gradient Echo (MPRAGE) sequence with the following parameters: sagittal slice thickness, 1.0 mm, no gap, repetition time (TR), 2300.0 msec, Echo Time (TE), 2.98 msec, flip angle, 9°; Inversion Time (TI) of 900 msec, and imaging matrix size, 256 × 240 × 176.

### 2.5. Gray Matter Volume and Curve Index Measurements

Then, 3D Slicer (http://www.slicer.org accessed on 23 March 2022, Surgical Planning Laboratory, Harvard University, Boston, MA, USA, Version 4.11) and FreeSurfer (http://www.freesurfer.net accessed on 23 March 2022, Laboratory for Computational Neuroimaging at the Athinoula A. Martinos Center for Biomedical Imaging, Boston, MA, USA, Version 7.1.1) softwares were used to measure the volume and curve index of the gray matter for each subcortical region from the 3D T1 image data in DICOM (Digital Imaging and Communications in Medicine) format [14]. The DICOM files were converted into NifTI (Neuroimaging Informatics Technology Initiative) format using 3D Slicer, and then reconstructed into a 2D cortical surface using the recon-all function of FreeSurfer [15,16]. The reconstruction involved several steps of the recon-all function, including motion correction, skull stripping, normalization and transformation, white and gray matter segmentation, averaging and smoothing, parcellation of subcortical regions, and measurement of parcellation statistics. The results of the parcellation statistics provided the gray matter volume and curvature index for each of the 34 subcortical regions per hemisphere.

### 2.6. Statistical Analysis

Baseline characteristics based on the data are presented as mean standard deviation (SD) for continuous variables and percentages for categorical variables. Differences between the H-MMSE and L-MMSE groups were confirmed using the Student’s *t*-test for continuous variables and chi-square tests for categorical variables. The relationship between gait characteristics and the MCI stage was evaluated using multiple logistic regression analysis. We ran three regression models. In model 1, we performed a binary logistic regression analysis with each gait parameter as the determinant and the MCI stage as the outcome variable. In model 2, we performed a multiple logistic regression analysis with age, sex, years of education, and each gait parameter. In model 3, we performed a multiple logistic regression analysis with age, sex, years of education, number of comorbidities, BMI, and gait parameters. 

Disease risk is expressed as odds ratios (OR) with 95% confidence interval (95% CI). Statistical significance was defined as *p* < 0.05. All statistical analyses were performed using SPSS version 25 software (SPSS Inc., Chicago, IL, USA).

To evaluate the topography of the gray matter related to gait characteristics, linear regression analysis was performed on the voxel-based morphometry analysis in Python using the scikit-learn library after controlling for age, sex, year of education, number of comorbidities, BMI, and ICV. Correction for multiple comparisons was accomplished using a false discovery rate correction at a corrected probability value of 0.05. We determined that only values with a *t*-value of 5 or higher had a significant association; thus, only the results showing a *t*-value of 5 or higher are presented in the results.

## 3. Results

### 3.1. Demographics, Clinical Characteristics, and Gait Characteristics

Detailed demographic and clinical characteristics of the participants are presented in Table 1. The H-MMSE group had a higher proportion of females than the L-MMSE group. Years of education were shorter in the H-MMSE group than in the L-MMSE group. The H-MMSE group had a higher number of comorbidities than the L-MMSE group. The frequency according to the type of comorbidities was presented in Supplementary Table S1. Body mass index (BMI) was higher in the H-MMSE group than in the L-MMSE group.

Table 2 presents the gait characteristics of the participants. The L-MMSE group had larger H-H base support values than the H-MMSE group. 

### 3.2. Association between Gait Characteristics and Mild Cognitive Impairment

Table 3 shows the risk of L-MMSE according to gait characteristics. Patients with higher gait velocity had 0.96-fold odds of L-MMSE (OR 0.96, 95% CI 0.92–0.99). Step length was associated with L-MMSE, with a lower odds ratio for the left side (OR 0.81, 95% CI 0.71–0.91) than the right side (OR 0.86, 95% CI 0.78–0.95). H-H base support was associated with L-MMSE, with higher odds ratios for the left (OR 1.29, 95% CI 1.08–1.58) than the right side (OR 1.39, 95% CI 1.11–1.75). Patients with greater step length variability on the right side had 1.22-fold odds of L-MMSE (OR 1.22, 95% CI 1.03–1.44). A shorter swing phase, longer stance phase, and longer double support phase were also associated with L-MMSE.

Decreased and distorted gray matter areas are associated with gait characteristics related to cognitive dysfunction.

**Figure 1:**
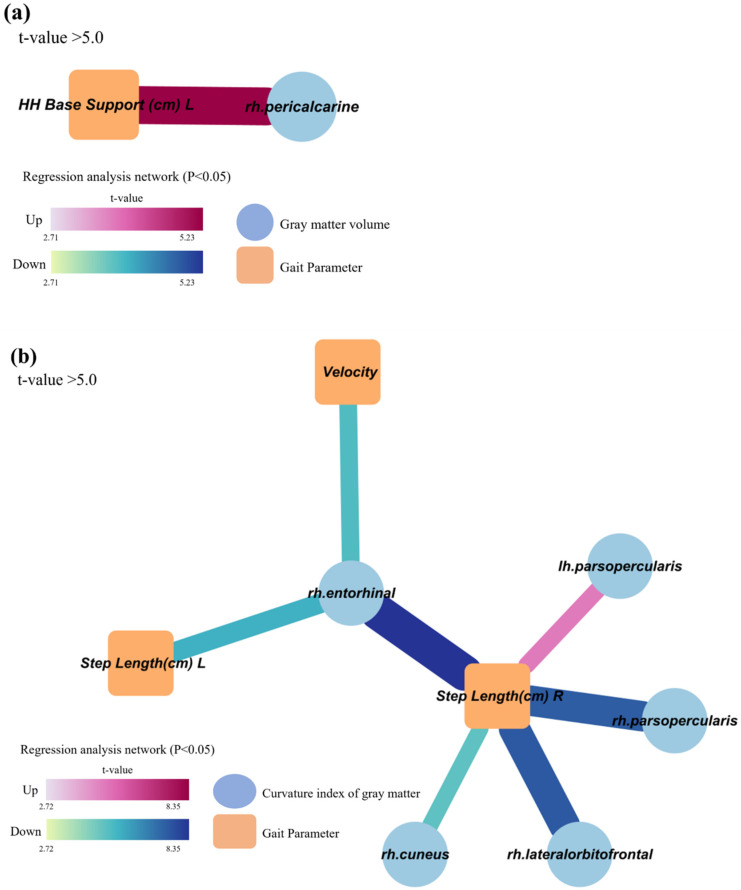
Regional areas associated with gait characteristics related to cognitive dysfunction. (**a**) Regional volume change of gray matter in the right pericalcarine area was associated with gait characteristics related to cognitive dysfunction. (**b**) Regional distortion of gray matter in the right entorhinal, right lateral orbitofrontal, right cuneus, and right and left pars opercularis areas was associated with gait characteristics related to cognitive dysfunction. We displayed the results with a *t*-value > 5 in the figure. “Up” signifies a positive relationship and “down” signifies a negative relationship. The thickness of the line indicates the degree of the association. Abbreviations: HH, heel to heel; rh, right; lh, left; L, left.

Figure 1 shows the regional areas associated with gait characteristics. A regional decrease in gray matter volume is associated with gait characteristics. Increased H-H base support is associated with the right pericalcarine area.

Regional distortion of the gray matter is also associated with gait characteristics. Slowed gait velocity was associated with an increased gray matter curvature index in the right entorhinal area. An increased step length was associated with an increased curvature index of the gray matter in the right entorhinal, right lateral orbitofrontal, right cuneus, and right pars opercularis areas. An increased step length was also associated with a decreased curvature index of the gray matter in the left pars opercularis.

## 4. Discussion

Slower gait velocity, shorter step length, wider H-H base support, shorter swing phase, longer stance phase, and longer double support were all associated with L-MMSE, as well as increased step length variability. Subsequently, we investigated the brain regions associated with these gait characteristics in relation to L-MMSE using MRI. We found that a decrease in gray matter volume in the right pericalcarine areas was associated with gait characteristics related to L-MMSE. Additionally, an increase in the curvature of gray matter in the right entorhinal, right lateral orbitofrontal, and right pars opercularis areas was associated with gait characteristics related to L-MMSE.

In this study, we found that slower gait was associated with L-MMSE, which is consistent with the findings of previous studies suggesting that motor impairment occurs during the course of cognitive decline [11]. In previous studies, patients with MCI showed decreased motor function compared with those without cognitive impairment [17,18]. Patients with MCI were also more likely to exhibit parkinsonian features such as bradykinesia, rigidity, and gait dysfunction [19,20]. These parkinsonian features were associated with the severity of cognitive impairment [19]. However, it is not clear what causes motor function to decrease in patients with MCI. Some studies have shown that the cognitive function in a specific area is related to gait speed. A previous study found that slow gait speed was associated with lower executive function [21], and another longitudinal study reported that low memory and executive functions were related to gait speed [22]. In studies using neuroimaging studies, some researchers have reported that cerebrovascular lesions, such as periventricular white matter changes in the frontal lobe, are associated with gait disturbances in patients with MCI [18]. On the other hand, some researchers have reported that more parkinsonian features are seen in patients with MCI than in cognitively normal older adults, regardless of the vascular burden [19]. Also, in another study, temporal lobe atrophy was correlated with poor mobility, independent of cerebrovascular disease [23].

We reported that shorter step length, wider H-H base support, shorter swing phase, longer stance phase, longer double support, and increased step length variability were associated with L-MMSE. Since these characteristics can appear when there is a problem with balance function [24] the gait characteristics in our study suggest that MCI patients with more severe cognitive impairment had lower balance function. In a previous study, patients with MCI had decreased balance function in the eyes-open balance test but not in the eyes-closed balance test [3]. They suggested that visual information had to be processed to maintain balance with eyes open [25] but patients with MCI had a decreased ability to process visual information [26] leading to a more pronounced balance function impairment when their eyes were opened. Another study showed that impaired executive function, including attention, was associated with impaired balance in patients with cognitive impairment, and that the decrease in balance function was more pronounced with the severity of cognitive impairment [27].

Step length variability is also associated with L-MMSE. Similar to our findings, a previous study reported that gait variability was greater in patients with dementia [28]. Gait stability has been reported to be highly related to balance [29] and another report has suggested that it is highly related to executive function, including attention [28]. Although gait is a skilled movement, it is generally believed that no cognitive function is involved [30]. However, even for previously skilled movements, executive functions that integrate attention with motor control and input from higher cortical motor centers are required to maintain gait. Gait stability may be impaired in patients with cognitive impairment because these functions are impaired [31].

A decrease in the volume of gray matter in the right pericalcarine area was associated with gait characteristics related to L-MMSE. An increase in the curvature of the gray matter in the right cuneus was also associated with the gait characteristics. The pericalcarine gyrus (PCG) plays an important role in the visual networks [32]. Considering that low gait function is related to a decrease in gray matter volume in these areas, visual function seems to play an important role in maintaining gait function. Similar to this study, brain regions, such as the pericalcarine gyrus, were related to gait function in a study on the functional connectivity of brain regions related to gait function in patients with multiple sclerosis [33]. In addition, the cuneus is generally an area involved in basic visual processing, and it is also involved in locomotion. In one case report, gait dyspraxia was reported after an infarction in the right cuneus [34]. In our study, we found that an increase in the curvature index of the right cuneus was associated with a decrease in step length, which can be interpreted in the same context as previous studies.

In this study, an increase in the curvature of gray matter in the right entorhinal, right lateral orbitofrontal, and right cuneus regions was associated with gait characteristics. The entorhinal cortex is the main input and output structure of the hippocampal formation, connecting the cortex and hippocampus [35]. In a pathologic study, the pathological changes associated with Alzheimer’s disease first occurred in the entorhinal cortex, and the change started there and then spread to the hippocampus and cortex [36]. In a clinical study of the hippocampal structures, the entorhinal cortex best reflected disease progression in patients with MCI [37]. In previous studies, hippocampal volume was associated with gait and cognitive function in patients with MCI [37,38] and the entorhinal cortex itself has been associated with gait disturbance in previous studies [37]. The hippocampus and the prefrontal cortex are functionally connected through the entorhinal cortex, and the hippocampus also has a function in the integration of sensory and motor information [39]. Although our study did not show a decrease in the hippocampal volume in relation to gait characteristics, a deformity of the entorhinal cortex was observed. Since the entorhinal cortex is a region that better reflects disease progression than the entire hippocampus [37], we speculated that the deformity might occur earlier than in other regions in relation to gait disturbance in patients with MCI.

Deformities of the pars opercularis and lateral orbitofrontal areas were also observed in this study. Dysfunction of the prefrontal cortex, including the orbitofrontal cortex and the pars opercularis, plays a major role in gait dysfunction in Alzheimer’s disease (AD). The orbitofrontal cortex plays an important role in inhibitory control [40]. Executive-attention function, including inhibition control, is impaired in patients with prodromal AD before memory loss and is also involved in gait function [41,42]. In a previous aging study, gait function was associated with amyloid deposition in the orbitofrontal area [43]. The pars opercularis belongs to Broca’s area and is the location of the frontal part of the mirror neurons. Mirror neurons play an important role in the imitation and execution of actions. Frontal mirror neurons are known to exert greater activity during execution [44].

This study had several limitations. First, we did not classify the patients with amnestic mild cognitive impairment. Therefore, we cannot exclude the possibility that participants with degenerative pathology related to parkinsonism or vascular damage may have been present. Secondly, musculoskeletal problems affecting gait disturbances were not considered. Third, as this was a cross-sectional study, it was difficult to establish causal relationships. Fourth, due to the diverse comorbidities among the patients, there is a possibility that these comorbidities may have affected brain function and cognitive abilities. While there was no statistically significant difference between the two groups, considering that the patients’ comorbidities include previous stroke or depression, it is not clear whether the poorer gait characteristics in the lower MMSE group were exclusively due to cognitive decline or if they were a result of various comorbidities affecting cognitive function. Fifth, this study defines patients with a CDR score of 0.5 as mild cognitive impairment patients, which means that patients with relatively broad range of MMSE scores are also included. Consequently, since our study targets a heterogeneous population of MCI patients, caution is required in interpreting the results. However, we investigated gait characteristics related to cognitive dysfunction and identified cortical areas related to gait characteristics in patients with MCI. In the future, more research is needed to determine whether the gait characteristics identified in our study will be helpful for the early detection of MCI.

## Figures and Tables

**Table 1:** Demographic and clinical characteristics.

	Total (n = 80)	H-MMSE (n = 53)	L-MMSE (n = 27)	*p* Value
Mean age	74.6 ± 5.74	74.6 ± 5.33	74.6 ± 6.58	0.95
Gender, female (%) ^a^	58 (72.5)	43 (81.1)	15 (55.6)	0.020
Year of education	6.88 ± 3.980	5.94 ± 4.069	8.72 ± 3.114	0.003
Number of comorbidities	3.7 ± 1.70	3.9 ± 1.61	3.1 ± 1.77	0.047
MMSE	23.0 ± 3.70	24.2 ± 3.26	20.5 ± 3.26	<0.0001
Depression scale	4.9 ± 4.09	4.9 ± 4.24	5.1 ± 3.83	0.833
Anxiety scale	6.8 ± 6.52	7.1 ± 6.87	6.3 ± 5.86	0.600
QOL scale	34.4 ± 8.67	33.3 ± 8.39	36.5 ± 9.00	0.124
Stress scale	1.57 ± 0.664	1.62 ± 0.716	1.49 ± 0.551	0.423
Height	156.1 ± 8.11	155.4 ± 7.98	157.7 ± 8.30	0.232
Weight	61.0 ± 10.92	61.9 ± 11.47	59.3 ± 9.72	0.324
BMI, mean (SD)	25.0 ± 3.70	25.6 ± 3.84	23.8 ± 3.17	0.045
Waist circumference (cm)	87.8 ± 10.12	87.6 ± 10.39	88.1 ± 9.75	0.825
Systolic BP (mmHg)	128.6 ± 18.27	130.0 ± 18.44	126.0 ± 17.97	0.349
Diastolic BP (mmHg)	77.0 ± 9.82	77.4 ± 9.41	76.1 ± 10.73	0.606
Fasting glucose (mg/dL)	108.8 ± 26.28	111.3 ± 28.44	104.0 ± 21.08	0.240
Total cholesterol (mg/dL)	162.4 ± 32.54	162.1 ± 34.00	163.0 ± 30.11	0.913

Data are mean ± SD unless otherwise indicated. ^a^ Number (%). Abbreviations: QOL, quality of life; BMI, body mass index; BP, blood pressure; SD, standard deviation.

**Table 2:** Gait characteristics.

	Total (n = 80)	H-MMSE (n = 53)	L-MMSE (n = 27)	*p* Value
Gait velocity	93.6 ± 20.16	93.9 ± 21.48	90.9 ± 17.35	0.404
Cadence	107.1 ± 11.52	106.4 ± 12.02	108.3 ± 10.58	0.498
Step time (sec)				
Left	0.6 ± 0.07	0.6 ± 0.07	0.6 ± 0.06	0.575
Right	0.6 ± 0.07	0.6 ± 0.07	0.6 ± 0.05	0.348
Step length (cm)				
Left	51.8 ± 8.52	52.9 ± 8.60	49.7 ± 8.09	0.111
Right	52.3 ± 8.27	53.0 ± 8.15	50.9 ± 8.47	0.268
Cycle time (sec)				
Left	1.1 ± 0.13	1.1 ± 0.15	1.1 ± 0.11	0.418
Right	1.1 ± 0.13	1.1 ± 0.15	1.1 ± 0.11	0.468
H-H base support (cm)				
Left	9.0 ± 3.12	8.5 ± 3.14	10.1 ± 2.82	0.027
Right	8.8 ± 2.99	8.1 ± 2.78	10.2 ± 2.95	0.002
Swing % of cycle				
Left	36.8 ± 2.28	36.9 ± 2.23	36.7 ± 2.42	0.776
Right	36.6 ± 2.45	36.9 ± 2.70	35.9 ± 1.75	0.085
Stance % of cycle				
Left	63.2 ± 2.28	63.1 ± 2.23	63.3 ± 2.42	0.795
Right	63.4 ± 2.46	63.1 ± 2.70	64.1 ± 1.74	0.051
Double support % of cycle				
Left	26.4 ± 4.20	26.0 ± 4.41	27.2 ± 3.72	0.242
Right	26.6 ± 4.41	26.2 ± 4.74	27.2 ± 3.67	0.354
Step time variability (%)				
Left	4.1 ± 2.77	4.1 ± 2.72	4.2 ± 2.91	0.849
Right	3.7 ± 2.56	3.5 ± 2.76	4.1 ± 2.10	0.318
Step length variability (%)				
Left	2.4 ± 1.65	2.3 ± 1.45	2.6 ± 2.00	0.400
Right	4.8 ± 3.45	4.4 ± 3.43	5.5 ± 3.45	0.169

**Table 3:** Gait parameters and odds of cognitive impairment.

	Model 1		Model 2		Model 3	
	OR (95% CI)	*p* Value	OR (95% CI)	*p* Value	OR (95% CI)	*p* Value
Gait velocity	0.990 (0.967, 1.013)	0.400	0.968 (0.940, 0.998)	0.035	0.957 (0.924, 0.991)	0.015
Cadence	1.015 (0.973, 1.057)	0.493	1.008 (0.959, 1.059)	0.763	1.011 (0.955, 1.069)	0.712
Step time (sec)						
Left	0.135 (0.000, 136.191)	0.570	0.700 (0.000, 2222.031)	0.931	0.896 (0.000, 8368.103)	0.981
Right	0.025 (0.000, 53.164)	0.347	0.083 (0.000, 687.854)	0.589	0.069 (0.000, 2747.780)	0.621
Step length (cm)						
Left	0.955 (0.901, 1.011)	0.114	0.878 (0.805, 0.958)	0.003	0.805 (0.711, 0.912)	0.001
Right	0.968 (0.913, 1.025)	0.266	0.894 (0.822, 0.972)	0.009	0.861 (0.781, 0.950)	0.003
Cycle time (sec)						
Left	0.213 (0.005, 8.789)	0.415	0.504 (0.007, 38.928)	0.757	0.567 (0.004, 83.640)	0.824
Right	0.252 (0.006, 10.106)	0.464	0.475 (0.006, 37.650)	0.739	0.496 (0.003, 75.879)	0.785
H-H base support (cm)						
Left	1.192 (1.015, 1.400)	0.033	1.167 (0.978, 1.392)	0.087	1.288 (1.047, 1.584)	0.017
Right	1.292 (1.083, 1.542)	0.004	1.266 (1.040, 1.541)	0.019	1.391 (1.108, 1.746)	0.005
Swing % of cycle						
Left	0.971 (0.792, 1.189)	0.773	0.864 (0.684, 1.091)	0.218	0.724 (0.541, 0.967)	0.029
Right	0.844 (0.694, 1.027)	0.090	0.716 (0.560, 0.916)	0.008	0.539 (0.378, 0.768)	0.001
Stance % of cycle						
Left	1.028 (0.839, 1.260)	0.792	1.154 (0.913, 1.458)	0.230	1.377 (1.031, 1.839)	0.030
Right	1.183 (0.973, 1.439)	0.092	1.392 (1.089, 1.779)	0.008	1.845 (1.298, 2.621)	0.001
Double support % of cycle						
Left	1.069 (0.956, 1.194)	0.242	1.161 (1.014, 1.329)	0.030	1.357 (1.155, 1.641)	0.002
Right	1.051 (0.946, 1.167)	0.352	1.149 (1.009, 1.308)	0.036	1.354 (1.130, 1.623)	0.001
Step time variability						
Left	1.017 (0.860, 1.201)	0.847	1.022 (0.854, 1.222)	0.815	1.045 (0.844, 1.295)	0.684
Right	1.095 (0.915, 1.311)	0.320	1.147 (0.942, 1.397)	0.173	1.188 (0.963, 1.465)	0.109
Step length variability						
Left	1.143 (0.865, 1.512)	0.347	1.178 (0.863, 1.609)	0.302	1.166 (0.845, 1.609)	0.350
Right	1.098 (0.961, 1.255)	0.170	1.149 (0.986, 1.339)	0.076	1.215 (1.027, 1.436)	0.023

Model 1: Separate models associated each gait parameters with cognitive impairment. Model 2: Single model including age, sex, years of education, and each gait parameter. Model 3: Single model including age, sex, years of education, number of comorbidities, BMI, and each gait parameter.

## Data Availability

The data that support the findings of this study are available on request from the corresponding author. The data are not publicly available due to privacy or ethical restrictions.

## Supplementary Materials

The following supporting information can be downloaded at: https://www.mdpi.com/article/10.3390/jcm12165347/s1, Table S1: Frequencies of comorbidities. Table S2: Classification rates for linear regression analysis.
