# Supplementary material for: Relationship of Neural Correlates of Gait Characteristics and Cognitive Dysfunction in Patients with Mild Cognitive Impairment
Source: J Clin Med. 2023 Aug 17;12(16):5347. doi: 10.3390/jcm12165347 (PMC10455461; doi:10.3390/jcm12165347)
Supplement: Supplementary file 1 [file jcm-12-05347-s001.zip › Supplementary table.pdf]

Supplementary Table S1. Frequencies of comorbidities

	Total (n=80)	H-MMSE (n=53)	L-MMSE (n=27)	p value
Hypertension	44 (55.0)	32 (60.4)	12 (44.4)	0.132
Diabetes mellitus	22 (27.5)	14 (26.4)	8 (29.6)	0.479
Dyslipidemia	41 (51.3)	32 (60.4)	9 (33.3)	0.020
Cardiac disease	10 (18.9)	4 (14.8)	14 (17.5)	0.453
Previous stroke	5 (6.3)	5 (9.4)	0 (0.0)	0.119
Liver disease	6 (7.5)	3 (5.7)	3 (11.1)	0.324
Depression	23 (28.8)	13 (24.5)	10 (37.0)	0.182
Renal disease	9 (11.3)	8 (15.1)	1 (3.7)	0.122
Pulmonary disease	6 (7.5)	1 (1.9)	5 (18.5)	0.015
Arthritis	42 (52.5)	34 (64.2)	8 (29.6)	0.003
Chronic gastrointestinal disease	26 (32.5)	18 (34.0)	8 (29.6)	0.449
Cancer	7 (8.8)	4 (7.5)	3 (11.1)	0.439
Previous fracture	26 (32.5)	19 (35.8)	7 (25.9)	0.262
Other disease	23 (28.8)	16 (30.2)	7 (25.9)	0.450

Other disease included thyroid disease, osteoporosis, and previous history of minor surgery, such as cataract operation.

Data are Number (%)

Supplementary Table S2. Classification rates for linear regression analysis

	Model 1	Model 2	Model 3
Gait velocity	66.3%	71.3%	73.8%
Cadence	66.3%	67.5%	67.5%
Step time (sec)			
Left	66.3%	67.5%	70.0%
Right	66.3%	67.5%	68.8%
Step length (cm)			
Left	66.3%	72.5%	78.8%
Right	65.0%	66.3%	73.8%
Cycle time (sec)			
Left	66.3%	67.5%	70.0%
Right	66.3%	67.5%	70.0%
H-H base support (cm)			
Left	67.5%	72.5%	75.0%
Right	70.0%	73.8%	80.0%
Swing % of cycle			
Left	66.3%	68.8%	73.8%
Right	62.5%	68.8%	81.3%
Stance % of cycle			
Left	66.3%	68.8%	73.8%
Right	62.5%	70.0%	81.3%
Double support % of cycle			
Left	63.8%	71.3%	75.0%
Right	65.0%	67.5%	80.0%
Step time variability			
Left	66.3%	67.5%	68.8%
Right	63.8%	63.8%	71.3%
Step length variability			
Left	67.5%	65.0%	66.3%
Right	63.8%	65.0%	71.3%
